# RACK(1) to the future – a historical perspective

**DOI:** 10.1186/1478-811X-11-53

**Published:** 2013-08-01

**Authors:** Dorit Ron, David R Adams, George S Baillie, Aideen Long, Rosemary O’Connor, Patrick A Kiely

**Affiliations:** 1 The Gallo Research Center, Department of Neurology, University of California San Francisco, San Francisco, CA 94143, USA; 2 Department of Chemistry, Heriot-Watt University, Riccarton Campus, Edinburgh EH14AS, Scotland, UK; 3 Institute of Cardiovascular & Medical Science, College of Medical, Veterinary & Life Sciences, University of Glasgow, Glasgow G12 8QQ, UK; 4 Clinical Medicine, Trinity College Dublin, Dublin, Ireland; 5 Cell Biology Laboratory, Department of Biochemistry, BioSciences Institute, University College Cork, Cork, Ireland; 6 Department of Life Sciences, Materials and Surface Science Institute and Stokes Institute, University of Limerick, Limerick, Ireland

## Abstract

This perspective summarises the first and long overdue RACK1 meeting held at the University of Limerick, Ireland, May 2013, in which RACK1’s role in the immune system, the heart and the brain were discussed and its contribution to disease states such as cancer, cardiac hypertrophy and addiction were described. RACK1 is a scaffolding protein and a member of the WD repeat family of proteins. These proteins have a unique architectural assembly that facilitates protein anchoring and the stabilisation of protein activity. A large body of evidence is accumulating which is helping to define the versatile role of RACK1 in assembling and dismantling complex signaling pathways from the cell membrane to the nucleus in health and disease. In this commentary, we first provide a historical perspective on RACK1. We also address many of the pertinent and topical questions about this protein such as its role in transcription, epigenetics and translation, its cytoskeletal contribution and the merits of targeting RACK1 in disease.

## Historical perspective

It has been 20 years since RACK1 was cloned as the first identified binding protein for Protein Kinase C (PKC), and the road the Mochly-Rosen group took toward its discovery was not trivial. The role of scaffolding proteins in the temporal and spatial regulation of signal transduction seems obvious today, however, this was not the case in the late 1980s when Prof. Daria Mochly-Rosen developed the hypothesis that anchoring/scaffolding proteins control the specificity of substrate phosphorylation and function of Protein Kinase C (PKC) isozymes. An alpha phage display library/overlay assay strategy was used to identify binding proteins that interact with active PKC isoforms. To this day Dr. Ron remembers when one gene product was identified and was termed RACK1 for Receptor for Activated C Kinase [1]. Using very primitive bioinformatics programs, RACK1 was identified as a WD-40 motif protein with high homology to the beta subunit of G protein (G-beta) [1]. Over the years, many excellent studies expanded the role of RACK1 as a true scaffolding protein that interacts with a large, diverse group of proteins and by doing so, contributes to the regulation of various signaling cascades and biological functions (for a recent review see [2]). Therefore, it is not entirely surprising that the amino acid sequence of RACK1 is highly conserved throughout evolution, that the protein is highly expressed in all mammalian cells, and that a global deletion of the RACK1 gene is embryonically lethal [3]. Other unique characteristics of this fascinating protein are its ability to translocate from one cellular compartment to another, to form homo- and heterodimers with itself and with G-beta as well as with other scaffolding proteins such as 14-3-3zeta, and to contribute to translation and transcription events [2,4]. Finally, as described below a new frontier in RACK1 research is recently emerging as studies reveal that RACK1 interacts with several cytoskeletal proteins, thus suggesting a role in cell trafficking and morphology.

## RACK1 as a scaffolding protein

The conserved seven blade propeller structure of RACK1 facilitates the folding order into constituent propeller blades. These propeller blades are intrinsic to RACK1’s protein binding capacity and allow RACK1 to function as a signaling hub [2]. Comments are often directed at RACK1 biologists questioning the capacity of this protein to bind to diverse signaling and structural proteins. However, it is easy to forget that there is an estimated 100 million individual proteins involved in signal transduction pathways within a cellular milieu [5]. Scaffolding proteins, such as RACK1 organise proteins into linear and branching signaling networks and several scaffolding proteins can function within a particular signaling pathway [6]. For example, there are at least 10 scaffolding proteins that function within the tightly controlled MAPK pathway in mammalian cells [7]. Collectively, these scaffolding proteins are known to bind hundreds of different proteins. In its capacity, RACK1 is no different from other well studied scaffolding proteins such as Ras GTPase-activating-like protein (IQGAP1) and Beta-arrestin in its ability to bind many diverse signaling proteins [8-11]. Furthermore, when bound to scaffolding proteins, the local concentration of a signaling protein is increased. For this reason, it is essential that the scaffolds expression level is tightly controlled and even a slight deviation from the optimum expression level can have dramatic consequences for signaling networks and the cell.

## RACK1: a scaffolding protein with a central role in transcription, epigenetics & translation

RACK1 has a strong effect on transcription and translation by acting at critical points; principally the ribosomal small subunit and via nuclear translocation and regulation of chromatin and transcriptional complexes [2]. All available crystal structures of ribosomal 40S subunits include an accessible RACK1 molecule that can also bind to the translational initiation EIF3 complex and other signaling proteins [12-16]. Thus, RACK1 has potential to dock membrane-associated signaling complexes with translationally competent ribosomes to control spatial translation [2]. This is consistent with its scaffolding of Integrins with IGF-1R and Focal Adhesion signaling complexes [17-20] in cancer cells where local translation would facilitate migration. RACK1 may also regulate cellular responses to metabolic stress during translation. Its recruitment to RNA stress granules that form at translationally stalled mRNAs [21] is thought to prevent activation of stress kinase (p39, JNK) pathways and thereby allow cells to recover from stress rather than undergo apoptosis [22]. While transcription factors can be translationally controlled by RACK1, nuclear transcriptional complexes and chromatin may also be modulated by its association. This was demonstrated for the transcriptional response of the brain-derived neurotrophic factor (BDNF) in neurons. RACK1 becomes translocated to the nucleus in association with the scaffolding protein 14-33zeta in response to cAMP signaling to promote chromatin remodelling that includes H4 histone acetylation resulting in *BDNF* transcription [4,23]. This illustrates potential for RACK1 to be selectively translocated to the nucleus in response to cellular signals and suggests that RACK1 will be found within other transcriptional complexes as a critical mediator of dynamic cytoplasmic to nuclear signaling responses for environmental cues that control neuronal plasticity, epithelial cells migration or differentiation, and immune responses.

## RACK1 and cytoskeletal proteins: a new frontier

The cellular cytoskeleton, whose major components comprise of actin, microtubules and intermediate filaments, maintains cellular integrity and regulates multiple cellular functions including migration [24,25]. Localization of RACK1 at the cell membrane is consistent with its association with Beta-spectrin, a cytoskeletal protein that, as a heterodimer, forms a meshwork lining the intracellular side of the plasma membrane maintaining cytoskeletal structure and membrane integrity. This RACK1-Beta spectrin interaction facilitates PKCbeta localisation and represents a focal point for pleckstrin homology (PH) domain-containing substrates of this kinase [26]. RACK1 has also been shown to contribute to cell migration and is a crucial component and regulator of focal adhesion assembly/disassembly [17,27]. Focal adhesions are rich in cytoskeletal linker proteins including talin and paxillin which in turn are important regulators of the actin cytoskeleton. Through the regulation of Src kinase activity, RACK1 modulates paxillin phosphorylation, a key step in early adhesion formation during migration [28]. Earlier studies in yeast demonstrated that a RACK1 homolog Rkp1/Cpc2, regulates the integrity of the actin cytoskeleton during cell wall synthesis while in human platelets, RACK1 mediates the interaction between PKCbeta and alphaII/betaIII integrin, regulating actin cytoskeletal reorganisation and platelet spreading on fibrinogen [29,30]. Interestingly, in NIH 3 T3 cells, RACK1 was shown to inhibit Src-mediated p190RhoGAP signaling and actin cytoskeleton rearrangement [31]. Plectin is an intermediate filament (IF)-associated linker protein that also functions to regulate actin dynamics and serves as a scaffold for signaling proteins. RACK1-Plectin interactions are particularly important in recruiting and maintaining kinases at the IF cytoskeleton, regulating keratin architecture, adhesion and migration in epithelial cells [32,33]. The RACK1-Keratin interaction is also important in regulation of PKCalpha activity and stabilisation of desmosomes to control intercellular adhesion [34]. Non-mechanical cellular functions are also impacted by RACK1-Plectin mediated regulation of keratins and include the modulation of G1/S transition and MAP kinase activity [31,32]. Given the importance of phosphorylation and protein-protein interactions in the regulation of the cytoskeleton, further roles for RACK1 in this process are sure to emerge. In particular, the role of RACK1 in microtubule (MT) polymerisation, actin-MT interactions and regulation of cell polarity represent interesting candidates.

## RACK1 as a potential therapeutic target

As the number of binding partners and validated cellular functions for RACK1 has increased, so has its link with an array of disease states [2]. In many RACK1-associated pathological states, aberrant RACK1 signaling, which underpins the condition, arises from either increased or decreased expression of the scaffolding protein (reviewed in [2]). Presumably, sub-optimal protein levels of RACK1 interferes with its compartmentalisation and, in turn, hinder the formation of signaling complexes with correct stoichiometry at discrete intracellular locations. Cell permeable peptides that act to disrupt the interaction of a RACK1 “pool”, which associates with a single binding partner (PDE4D5), have been successful in inhibiting the metastasis and direction sensing of cancer cells without affecting the plethora of other vital RACK1 functions [27,35], Thus, it is reasonable to conclude that the therapeutic potential of RACK1 could be realised by small molecules that selectively target RACK1 interacting proteins. Indeed, two recent reports have showcased co-crystal structures of a RACK1-like, 7-bladed beta-propeller protein (WDR5 in this case) with small molecules that have been developed to interrupt binding of a single client protein [36,37]. These studies, along with the work of Orlicky *et al.*[38] are amongst the first describing beta-propeller/small molecule complexes. Interestingly, the report by Senisterra *et al.*[36], shows that a new small molecule can occupy the central channel on one face of the beta-propeller. For many beta-propellers this is a protein binding site, as is the case with the cyclin E phosphodegron binding to FBXW7, a component of E3ubiquitin ligase [39,40]. This research provides important precedent for the targeting of beta-propeller sites with small molecules and opens the way for protein-protein inhibitors for RACK1 that could potentially reverse the pathologies mentioned above.

## Concluding comments

There are, of course, numerous remaining questions that are of great interest. For example, how can one protein play such an important role in many and diverse biological functions? Is it possible that a number of RACK1 binding partners share common binding sites on RACK1? Is RACK1 function and/or expression levels regulated by posttranslational modifications such as phosphorylation, sumoylation and ubiquitination? Does RACK1 contribute to a large number of disease states and can RACK1 be used as a target for drug development? These are exciting times for RACK1 biologists. As more and more research areas converge on RACK1, we can expect answers to these questions to unfold. RACK1 biology would benefit greatly from detailed mechanistic mathematical modelling and quantitative experimentation to help us comprehend how RACK1 functions in systems biology, beyond its role as a scaffolding protein. We look forward to the next RACK1 conference, which we have no doubt will bring more exciting new data on the role of our favorite protein in cellular functions.

## Competing interests

The authors declare that they have no competing interests.
